# Crystal structure of (4*R*,5*S*,6*R*)-6-azido-5-benz­yloxy-3,3,4-tri­fluoro­azepan-1-ium 2,2,2-tri­fluoro­acetate from synchrotron data

**DOI:** 10.1107/S2056989015019416

**Published:** 2015-10-24

**Authors:** Alpesh Ramanlal Patel, Mohan M. Bhadbhade, Fei Liu

**Affiliations:** a Department of Chemistry and Biomolecular Sciences, Macquarie University, Sydney, NSW 2109, Australia; b Mark Wainwright Analytical Centre, The University of New South Wales, Sydney, 2052 NSW, Australia

**Keywords:** crystal structure, azepane, fluorination, fluorine *gauche* effect, hydrogen bonding

## Abstract

The structure of the title compound, C_15_H_16_F_6_N_4_O_3_, was determined using synchrotron radiation on an extremely small crystal (0.015 × 0.01 × 0.01 mm). Although the diffraction was weak, leading to high residuals and a poor data-to-parameter ratio, the data allowed ready solution and refinement to reveal the entire structure. The solid-state structure is in accordance with the absolute configuration assigned based on that of the known starting material. The compound comprises a highly substituted seven-membered N-heterocyclic cation and a tri­fluoro­methane­sulfonate counter-anion. The title compound crystallizes with two independent cations (*A* and *B*) and anions (*C* and *D*) in the asymmetric unit. Two geminal F atoms, a single F atom, a benzyl ether and an azide group are substituted on consecutive C atoms between the NH_2_ and CH_2_ units of the azepan-1-ium ring system. The seven-membered rings adopt different conformations with the principal differences occurring in the CF_2_CHFCH_2_ segments of the ring systems. The geminal F atoms on the quaternary C atom exhibit essentially identical bond angles [109 (2) and 106 (2)°] in the two independent mol­ecules. The two mol­ecules associate as a dimeric unit *via* two C—H⋯F inter­actions. An extensive series of N—H⋯O, N—H⋯F, C—H⋯O, C—H⋯N, C—H⋯F and C—H⋯π contacts generate a three-dimensional network with cations and anions linked into *ABCD* repeat columns along *a*.

## Abstract 2

This report describes the crystal structure of highly substituted tri­fluoro­azepane as an important building block of therapeutic inter­est. Data were collected with synchrotron radiation on a very small crystal.

## Chemical context   

Fluorine is virtually absent in naturally occurring bioactive mol­ecules. However, about 20% of pharmaceuticals and 30% of agrochemicals have at least one fluorine atom (Müller *et al.*, 2007 ▸; Isanbor & O’Hagan, 2006 ▸). Because fluorine is the most electronegative atom, it is small and forms very strong C—F bonds. The replacement of hydrogen by the bioisosteric fluorine in pharmacophores can lead to improved physical, chemical and biological properties (Ritter, 2012 ▸; Bégué & Bonnet-Delpon, 2006 ▸; Kirk, 2006 ▸).

Substituted azepane rings are prevalent in many bioactive natural compounds (Wipf & Spencer, 2005 ▸; Núñez-Villanueva *et al.*, 2011 ▸). Recently, substituted azepane rings and related compounds (imino­cyclitols or imino­sugars) have attracted considerable attention from medicinal chemists because of their great potential as glycosidase inhibitors (Stütz, 1999 ▸) and anti­diabetic (Pa­inter *et al.*, 2004 ▸), anti­cancer (Zitzmann *et al.*, 1999 ▸) and anti­viral agents (Laver *et al.*, 1999 ▸) and are also effective against HIV (Sinnott, 1990 ▸). The conformational control of such flexible ring structures is important to their bioactivity.

We have previously reported stereospecific de­oxy­fluorin­ation reactions of substituted seven-membered N-heterocycles such as azepanes (Patel & Liu, 2013 ▸, 2015 ▸; Patel *et al.*, 2013 ▸, 2014 ▸). The fluorine atoms that were added were found to regulate the conformational preferences of the N-heterocycle rings, and these fluorine-directed conformational changes were analysed by NMR techniques in solution in conjunction with computational modelling. Solution conformation analysis of the trifluorinated azepane was found to be difficult, and its direct solid-state structural analysis was also not feasible without having to add various substituents (Patel *et al.*, 2014 ▸). Incorporation of benz­yloxy and azide substituents in the 5- and 6-positions of the seven-membered ring led to crystal formation. However, the crystals were extremely small (0.015 × 0.01 × 0.01 mm) and diffraction data were obtained on the title trifluorinated azepane compound, C_15_H_16_F_6_N_4_O_3_ (**1**), directly using synchrotron radiation.
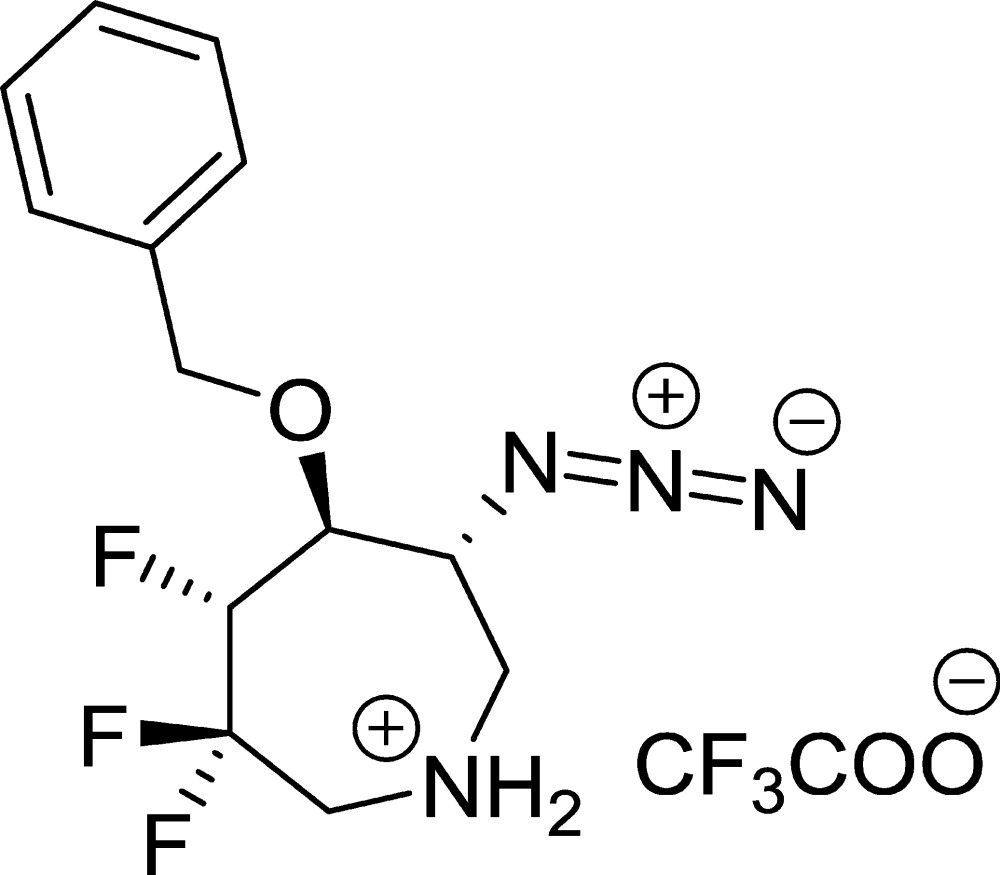


## Structural commentary   

**Figure 1:**
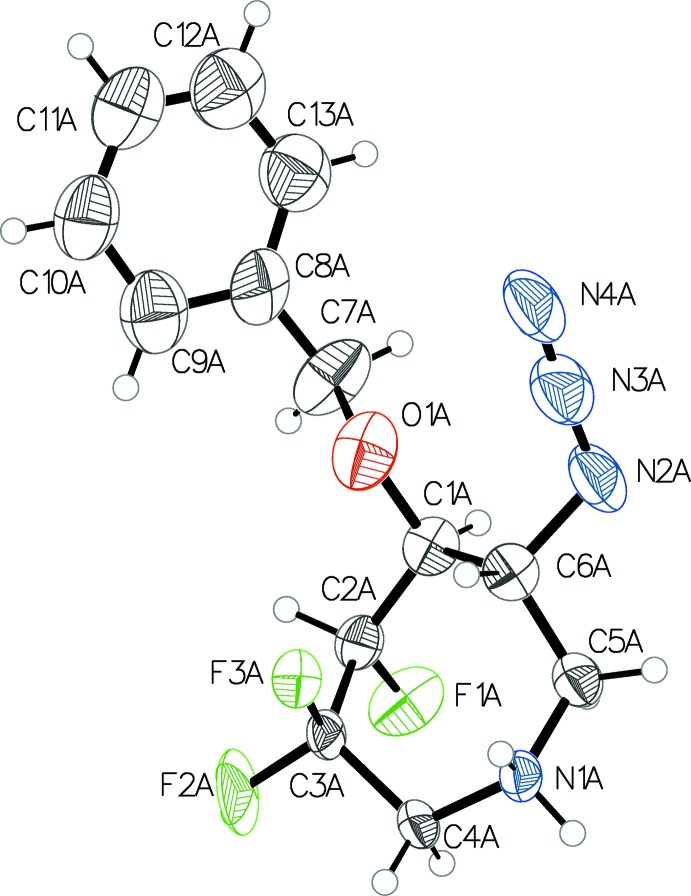
One of the two mol­ecules (*A*) in the asymmetric unit, showing the atom numbering. Displacement ellipsoids are drawn at the 50% probability level.

The compound crystallizes in a chiral space group (monoclinic, *P*2_1_) with two sets of cations and anions (mol­ecule *A* and *B*) in the asymmetric unit. Each cation has the same stereochemistry. An ORTEP view of the cation in mol­ecule *A*, Fig. 1 ▸, depicts the absolute configuration and atom-labelling scheme. The *B* cation and anion are labelled similarly but with trailing *B* characters after the atom numbers. The absolute configuration was assigned based on that of the known starting material.

**Figure 2:**
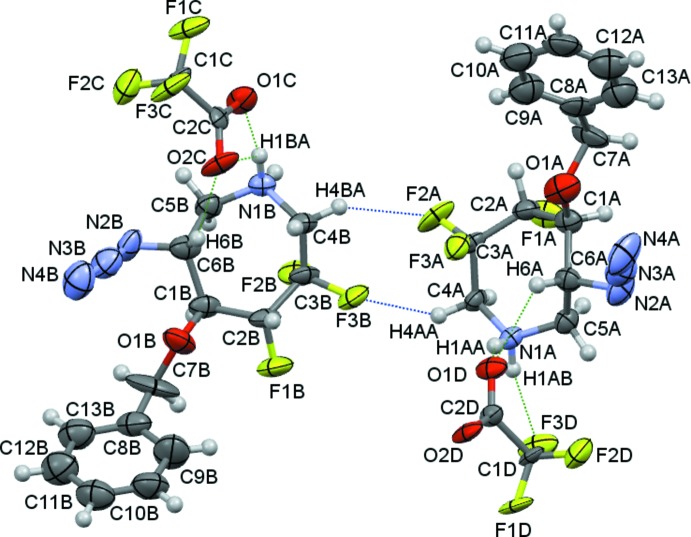
A view of the complete asymmetric unit consisting of two mol­ecules of (**1**) and two tri­fluoro­methane­sulfonate anions. In this and subsequent figures, hydrogen bonds are drawn as dashed lines.

An alternative ORTEP view, Fig. 2 ▸, shows the asymmetric unit with association between *A* and *B* mol­ecules *via* two C—H⋯F inter­actions to form dimers. The asymmetric unit is completed by the two triflate anions *C* and *D*. These are variously linked in an *A* to *C* and *B* to *D* fashion by N—H⋯O, N—H⋯F, C—H⋯O and C—H⋯F hydrogen bonds, Table 1 ▸.

**Figure 3:**
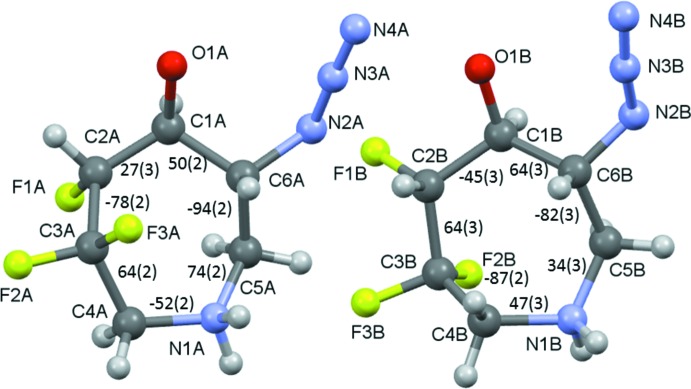
Conformations and torsion angles of the seven-membered rings of mol­ecules *A* and *B*.

The two mol­ecules differ significantly in their seven-membered ring conformations, in particular around C2 and C3 with significantly different torsion angles, Fig. 3 ▸, where the mol­ecules are involved in making dimeric contacts. Torsion angles within the two rings are shown in Fig. 3 ▸.

## Ring conformation analysis   

**Figure 4:**
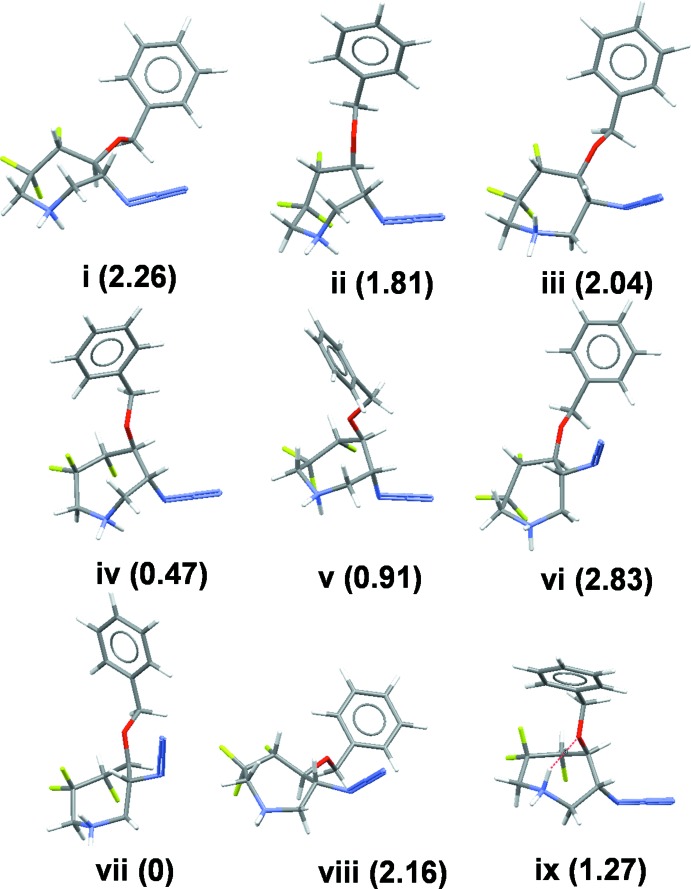
Nine conformations of compound (**1**) found by computational analysis. The number in parenthesis is the relative energy in kcal mol^−1^.

A computational analysis of ring conformations of compound (**1**) was carried out using protocols reported earlier (Patel *et al.*, 2013 ▸, 2014 ▸). Conformers were first generated by the stochastic method and minimized in the MMFF94x force field with chloro­form as the solvent to produce nine conformational clusters within 3–5 kcal mol^−1^ in energy that are distinct in their azepane-ring conformations, Fig. 4 ▸. Representative conformers were then subjected to DFT geometry optimization [SV(P) basis set at the B3LYP level in COSMO solvent chloro­form]. Two of the nine ring geometries (geometries vi and vii, Fig. 4 ▸) found by this computational analysis matched to geometries *A* and *B* of compound (**1**) in the unit cell, respectively. Hence the X-ray structure reported here for (**1**) validates our conformational analysis methodology as reported earlier (Patel *et al.*, 2013 ▸, 2014 ▸).

## Supra­molecular features   

**Figure 5:**
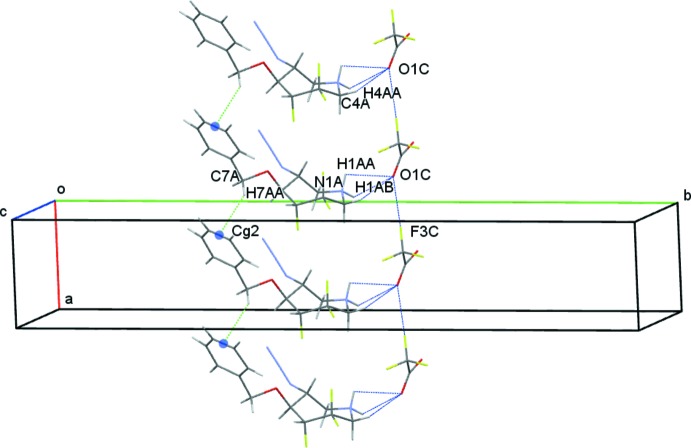
Inter­molecular contacts between *A* cations and *C* anions viewed along *c*. Midpoints of the C10*A*—C11*A* bonds are shown as coloured spheres.

**Figure 6:**
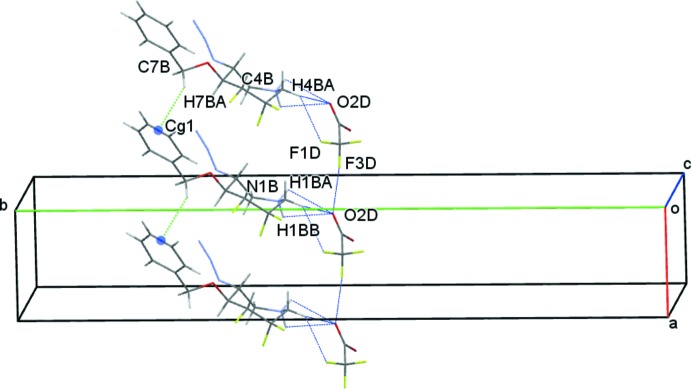
Inter­molecular contacts between *B* cations and *D* anions viewed along *c*. Midpoints of the C10*B*—C11*B* bonds are shown as coloured spheres.

**Figure 7:**
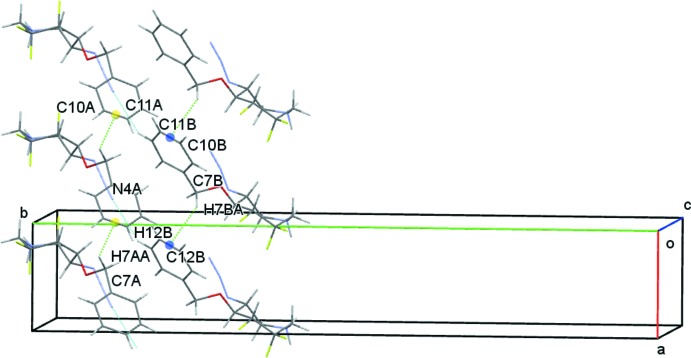
Inter­molecular contacts between the *A* and *B* cations viewed along *c*. Mid-points of the C10*A*—C11*A* and C10*B*—C11*B* bonds are shown as coloured spheres.

**Figure 8:**
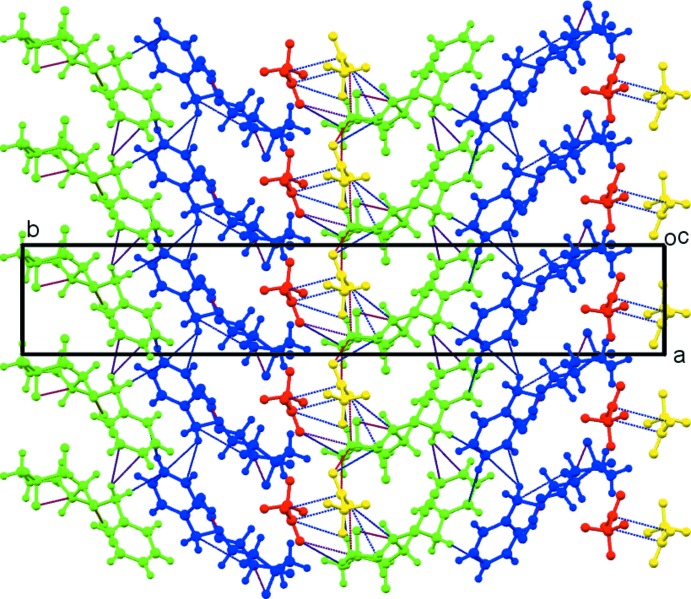
Packing of mol­ecules in the unit cell viewed along *c*. Mol­ecules *A* (green) and *B* (blue), tri­fluoro­methane­sulfonate anions *C* (red) and *D* (yellow). Hydrogen-bonding contacts are shown as dashed lines.

In the crystal structure, *C* anions form chains along the *a*-axis direction through F3*C*⋯O1*C* contacts at a distance of 2.78 (2) Å. Each anion further connects to an *A* cation with O1*C* accepting three interactions and N1*A* as a bifurcated donor, leading to the formation of N1*A*—H1*AA*⋯O1*C*, N1*A*—H1*AB*⋯O1*C* and C4*A*—H4*AA*⋯O1*C* hydrogen bonds and generating 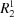
(4) and 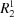
(5) ring motifs, respectively (Bernstein *et al.*, 1995 ▸). These contacts generate columns of *A* mol­ecules along *a*. These columns are further supported by weak C7*A*—H7*AA*⋯*Cg*2 contacts (*Cg*2 is the mid-point of the C10*A*—C11*A* bond of the C8*A–*C13*A* phenyl ring), Fig. 5 ▸. Similarly, *B* cations are linked to *D* anions with O2*D* accepting three interactions and forming N1*B—*-H1*BA*⋯O2*D*, N1*B*—H1*BB*⋯O2*D* and C4*B*—H4*BA*⋯O2*D* hydrogen bonds. Unlike the *AC* system however, a C4*B*—H4*BA*⋯F1*D* hydrogen bond completes the *B*⋯*D* cation–anion contacts. These generate 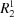
(4) and 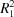
(5) ring motifs respectively. Weak C7*B*—H7*BA*⋯*Cg*1 contacts (*Cg*1 is the midpoint of the C10*B*–C11*B* bond of the C8*B–*C13*B* phenyl ring) link adjacent *B* mol­ecules, also forming columns of *B* cations and *D* anions along the *a-*axis direction, Fig. 6 ▸. Contacts between the *A* and *B* cations are limited to very weak C12*B*—H12*B*⋯N4*A* hydrogen bonds linking adjacent columns of *A* and *B* cations, Fig. 7 ▸. This eclectic mixture of contacts generates columns with an *ABCD* repeat unit in the direction of the *a* axis, Fig. 8 ▸. Additional N—H⋯O, C—H⋯O and C—H⋯F contacts result in a three-dimensional network of cations and anions stacked along *c*.

## Database survey   

A survey of the Cambridge Structural Database (Version 5.36, with three updates) (Groom & Allen, 2014 ▸) reveals the crystal structures of 11 unsubstituted azepanium (hexa­methyl­eneiminium) cations with a variety of counter-anions, see for example: Verlooy *et al.* (2010 ▸); Bakshi *et al.* (1994 ▸); Moritani *et al.* (1987 ▸); Kashino *et al.* (1981 ▸); Cameron & Scheeren (1977 ▸). Two of these salts also form co-crystals, Moritani & Kashino (2002 ▸); Misaki *et al.* (1989 ▸). However the structure of (3*R*,4*R*,5*S*,6*S*)-4,5,6-trihy­droxy-3-methyl azepanium chloride is the only one to be reported of a substituted azepanium salt, Li *et al.* (2008 ▸), highlighting the novelty of the present report.

## Synthesis and crystallization   

(4*R*,5*S*,6*R*)-6-Azido-5-benz­yloxy-3,3,4-tri­fluoro­azepane-1-carb­oxy­lic acid-*tert*-butyl ester (10 mg, 25.0 µ mol) was dissolved in tri­fluoro­acetic acid (TFA, 500 µL) at 298 K. The solution was allowed to stir for 5 min before the TFA was evaporated under an N_2_ flow. The reaction flask was kept under high vacuum (0.005 torr, 298 K) for 3 h to remove traces of TFA. A colorless, oily residue was obtained which was recrystallized from di­chloro­methane to give colorless needles characterized as (**1**) (10.0 mg, 97%). ^1^H NMR (600 MHz, CDCl_3_) *δ* 7.44–7.34 (*m*, 5H), 4.93 (*dd*, *J* = 44.19 (^1^ *J*_HF_), 14.7 Hz, 1H), 4.80 (*d*, *J* = 11.44 Hz, 1H), 4.73 (*d*, *J* = 11.44 Hz, 1H), 4.08 (*dd*, *J* = 8.71, 8.68 Hz, 1H), 3.89–3.82 (*m*, 1H), 3.67–3.57 (*m*, 2H), 3.48 (*d*, *J* = 14.0 Hz, 1H), 3.10 (*dd*, *J* = 14.0, 9.70 Hz, 1H); ^13^C NMR (150 MHz, CDCl_3_) *δ* 135.7, 129.0, 128.5, 128.5, 118.4 (*dd*, ^1^ *J*_CF_ = 247.66 Hz, ^2^ *J*_CF_ = 28.07 Hz), 90.2 (*ddd*, ^1^ *J*_CF_ = 186.03 Hz, ^2^ *J*_CF_ = 34.98 Hz, ^2^ *J*_CF_ = 27.82 Hz), 79.6 (dd, ^2^ *J*_CF_ = 24.93 Hz, ^3^ *J*_CF_ = 7.20 Hz), 73.9, 60.6, 45.8 (dd, ^2^ *J*_CF_ = 39.76 Hz, ^2^ *J*_CF_ = 25.66 Hz), 45.6.

## Refinement   

Crystal data, data collection and structure refinement details are summarized in Table 2 ▸. All H atoms were refined using a riding model, with N—H = 0.91 Å, C—H = 0.95 Å for aromatic, 1.00 Å for methine and 0.99 Å for methyl­ene, all with *U*_iso_(H) = 1.2*U*_eq_(N/C). Because of the lower reflections-to-parameter ratio, anisotropic displacement parameters of several atoms in the least-squares refinement had to be restrained using the RIGU command. These were applied to azide groups, atoms in the seven-membered and a few atoms in phenyl rings.

## Supplementary Material

Crystal structure: contains datablock(s) I, global. DOI: 10.1107/S2056989015019416/sj5470sup1.cif

Structure factors: contains datablock(s) I. DOI: 10.1107/S2056989015019416/sj5470Isup2.hkl

Supporting information file. DOI: 10.1107/S2056989015019416/sj5470Isup3.cml

CCDC reference: 1431203

Additional supporting information:  crystallographic information; 3D view; checkCIF report

## Figures and Tables

**Table 1:** Hydrogen-bond geometry (, ) *Cg*1 and *Cg*2 are the mid-points of the C10*A*C11*A* and C10*B*C11*B* bonds, respectively.

*D*H*A*	*D*H	H*A*	*D* *A*	*D*H*A*
N1*A*H1*AA*O1*D* ^i^	0.91	1.89	2.75(2)	156
N1*A*H1*AB*O2*D* ^ii^	0.91	1.79	2.677(19)	163
N1*B*H1*BA*O1*C* ^iii^	0.91	1.76	2.66(2)	170
N1*B*H1*BB*O2*C* ^iv^	0.91	1.85	2.75(2)	167
N1*A*H1*AA*O1*C*	0.91	2.57	2.88(3)	101
N1*A*H1*AB*O1*C*	0.91	2.57	2.88(3)	101
N1*B*H1*BB*O2*D* ^v^	0.91	2.63	2.94(3)	101
N1*B*H1*BA*O2*D* ^v^	0.91	2.70	2.94(3)	96
N1*A*H1*AB*F3*D* ^i^	0.91	2.60	3.010(16)	108
N1*B*H1*BB*F3*C* ^iv^	0.91	2.55	2.954(17)	108
C4*A*H4*AA*O1*C*	0.99	2.47	3.12(3)	122
C6*A*H6*A*O1*D* ^i^	1.00	2.42	3.14(3)	129
C4*B*H4*BA*O2*D* ^v^	0.99	2.39	3.08(3)	126
C6*B*H6*B*O2*C* ^iii^	1.00	2.37	3.22(3)	142
C12*B*H12*B*N4*A* ^vi^	0.95	2.71	3.42(4)	133
C4*A*H4*AA*F3*B*	0.99	2.50	3.29(3)	137
C4*A*H4*AA*F3*C* ^v^	0.99	2.69	3.27(3)	118
C5*A*H5*AA*F1*A*	0.99	2.59	3.18(2)	118
C5*A*H5*AB*F3*D* ^i^	0.99	2.70	3.31(2)	120
C6*A*H6*A*F3*A*	1.00	2.35	2.88(2)	112
C4*B*H4*BA*F2*A*	0.99	2.55	3.39(3)	142
C4*B*H4*BA*F3*D*	0.99	2.85	3.34(3)	111
C5*B*H5*BA*F2*B*	0.99	2.51	2.95(2)	107
C7*B*H7*BA*F1*B*	0.99	2.59	3.15(5)	116
C7*B*H7*BA* *Cg*1^v^	0.99	2.87	3.73(4)	146
C7*A*H7*AA* *Cg*2^v^	0.99	2.64	3.46(4)	140

Symmetry codes: (i) 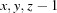; (ii) 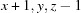; (iii) 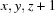; (iv) 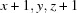; (v) 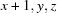; (vi) 
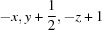
.

**Table 2:** Experimental details

Crystal data	
Chemical formula	C_13_H_16_F_3_N_4_O^+^C_2_F_3_O_2_
*M*_r_	414.32
Crystal system, space group	Monoclinic, *P*2_1_
Temperature (K)	100
*a*, *b*, *c* ()	5.8780(12), 34.503(7), 8.8120(18)
()	92.42(3)
*V* (^3^)	1785.6(6)
*Z*	4
Radiation type	Synchrotron, = 0.7293
(mm^1^)	0.16
Crystal size (mm)	0.015 0.01 0.01

Data collection	
Diffractometer	Bruker APEXII CCD
No. of measured, independent and observed [*I* > 2(*I*)] reflections	13709, 3642, 2175
*R*_int_	0.386
_max_ ()	21.5
(sin /)_max_ (^1^)	0.502

Refinement	
*R*[*F* ^2^ > 2(*F* ^2^)], *wR*(*F* ^2^), *S*	0.116, 0.261, 0.97
No. of reflections	3642
No. of parameters	505
No. of restraints	193
H-atom treatment	H-atom parameters constrained
_max_, _min_ (e ^3^)	0.56, 0.41

Computer programs: *XDS* (Kabsch, 2010 ▸), *SHELXS97* (Sheldrick, 2008 ▸), *SHELXL2014* (Sheldrick, 2015 ▸), *OLEX2* (Dolomanov *et al.*, 2009 ▸) and *Mercury* (Macrae *et al.*, 2008 ▸).

## supplementary crystallographic information

### Crystal data

**Table:**

C_13_H_16_F_3_N_4_O^+^·C_2_F_3_O_2_^−^	*Z* = 4
*M**_r_* = 414.32	*F*(000) = 848
Monoclinic, *P*2_1_	*D*_x_ = 1.541 Mg m^−^^3^
*a* = 5.8780 (12) Å	Synchrotron radiation, λ = 0.7293 Å
*b* = 34.503 (7) Å	µ = 0.16 mm^−^^1^
*c* = 8.8120 (18) Å	*T* = 100 K
β = 92.42 (3)°	Plate, colourless
*V* = 1785.6 (6) Å^3^	0.02 × 0.01 × 0.01 mm

### Data collection

**Table:**

Bruker APEXII CCD diffractometer	*R*_int_ = 0.386
ω scans	θ_max_ = 21.5°, θ_min_ = 2.4°
13709 measured reflections	*h* = −5→5
3642 independent reflections	*k* = −34→34
2175 reflections with *I* > 2σ(*I*)	*l* = −8→8

### Refinement

**Table:**

Refinement on *F*^2^	Hydrogen site location: inferred from neighbouring sites
Least-squares matrix: full	H-atom parameters constrained
*R*[*F*^2^ > 2σ(*F*^2^)] = 0.116	*w* = 1/[σ^2^(*F*_o_^2^) + (0.0001*P*)^2^] where *P* = (*F*_o_^2^ + 2*F*_c_^2^)/3
*wR*(*F*^2^) = 0.261	(Δ/σ)_max_ < 0.001
*S* = 0.97	Δρ_max_ = 0.56 e Å^−^^3^
3642 reflections	Δρ_min_ = −0.41 e Å^−^^3^
505 parameters	Absolute structure: Flack *x* determined using 390 quotients [(*I*^+^)-(*I*^-^)]/[(*I*^+^)+(*I*^-^)] (Parsons *et al.*, 2013)
193 restraints	Absolute structure parameter: 2.2 (10)

### Special details

**Table:**

Geometry. All e.s.d.'s (except the e.s.d. in the dihedral angle between two l.s. planes) are estimated using the full covariance matrix. The cell e.s.d.'s are taken into account individually in the estimation of e.s.d.'s in distances, angles and torsion angles; correlations between e.s.d.'s in cell parameters are only used when they are defined by crystal symmetry. An approximate (isotropic) treatment of cell e.s.d.'s is used for estimating e.s.d.'s involving l.s. planes.

### Fractional atomic coordinates and isotropic or equivalent isotropic displacement parameters (Å^2^)

**Table:**

	*x*	*y*	*z*	*U*_iso_*/*U*_eq_	
O1A	0.606 (3)	0.3889 (6)	0.6446 (15)	0.067 (5)	
F1A	1.1433 (19)	0.4338 (5)	0.6460 (13)	0.062 (4)	
F2A	0.923 (2)	0.4956 (5)	0.7835 (11)	0.080 (5)	
F3A	0.615 (2)	0.4787 (4)	0.6578 (10)	0.050 (3)	
N1A	0.835 (3)	0.4880 (5)	0.3749 (14)	0.035 (4)	
H1AA	0.6842	0.4943	0.3686	0.042*	
H1AB	0.9039	0.5018	0.3020	0.042*	
N2A	0.627 (4)	0.3894 (7)	0.3202 (19)	0.066 (6)	
N3A	0.444 (5)	0.3736 (8)	0.351 (2)	0.069 (7)	
N4A	0.278 (4)	0.3579 (9)	0.373 (2)	0.090 (9)	
C1A	0.794 (3)	0.4058 (7)	0.571 (2)	0.042 (5)	
H1A	0.9033	0.3848	0.5454	0.051*	
C2A	0.913 (4)	0.4343 (7)	0.668 (2)	0.044 (5)	
H2A	0.8938	0.4258	0.7751	0.053*	
C3A	0.844 (3)	0.4777 (7)	0.6604 (17)	0.034 (4)	
C4A	0.928 (3)	0.5013 (7)	0.5242 (17)	0.039 (5)	
H4AA	0.8869	0.5289	0.5379	0.046*	
H4AB	1.0967	0.4998	0.5247	0.046*	
C5A	0.855 (3)	0.4469 (7)	0.337 (2)	0.042 (5)	
H5AA	1.0121	0.4381	0.3625	0.050*	
H5AB	0.8243	0.4433	0.2270	0.050*	
C6A	0.691 (3)	0.4228 (7)	0.423 (2)	0.043 (5)	
H6A	0.5523	0.4384	0.4446	0.051*	
C7A	0.678 (4)	0.3547 (9)	0.737 (3)	0.070 (8)	
H7AA	0.7974	0.3618	0.8142	0.084*	
H7AB	0.7359	0.3339	0.6716	0.084*	
C8A	0.465 (4)	0.3422 (9)	0.810 (2)	0.062 (6)	
C9A	0.373 (5)	0.3642 (11)	0.921 (3)	0.081 (7)	
H9A	0.4346	0.3887	0.9491	0.097*	
C10A	0.178 (5)	0.3483 (11)	0.994 (3)	0.082 (7)	
H10A	0.1049	0.3630	1.0692	0.098*	
C11A	0.099 (5)	0.3121 (10)	0.955 (3)	0.079 (7)	
H11A	−0.0217	0.3010	1.0091	0.095*	
C12A	0.182 (5)	0.2932 (11)	0.848 (3)	0.084 (7)	
H12A	0.1102	0.2699	0.8148	0.101*	
C13A	0.375 (5)	0.3053 (11)	0.778 (3)	0.080 (7)	
H13A	0.4478	0.2887	0.7091	0.096*	
O1B	0.613 (3)	0.6932 (5)	0.8311 (16)	0.060 (4)	
F1B	0.916 (3)	0.6548 (5)	0.6524 (12)	0.079 (5)	
F2B	1.1956 (19)	0.6200 (4)	0.8697 (11)	0.055 (4)	
F3B	1.005 (2)	0.5828 (5)	0.7138 (11)	0.067 (4)	
N1B	0.961 (3)	0.5980 (6)	1.1146 (16)	0.036 (4)	
H1BA	0.8766	0.5850	1.1825	0.044*	
H1BB	1.1099	0.5922	1.1364	0.044*	
N2B	0.682 (3)	0.6932 (7)	1.1396 (17)	0.060 (6)	
N3B	0.485 (4)	0.7051 (7)	1.1158 (18)	0.067 (6)	
N4B	0.313 (4)	0.7214 (9)	1.101 (2)	0.081 (8)	
C1B	0.792 (4)	0.6723 (7)	0.891 (2)	0.045 (5)	
H1B	0.9306	0.6892	0.8960	0.054*	
C2B	0.830 (4)	0.6396 (7)	0.7792 (19)	0.046 (5)	
H2B	0.6767	0.6285	0.7498	0.055*	
C3B	0.983 (4)	0.6055 (7)	0.836 (2)	0.045 (5)	
C4B	0.898 (4)	0.5830 (8)	0.9578 (18)	0.044 (5)	
H4BA	0.9560	0.5562	0.9494	0.052*	
H4BB	0.7300	0.5819	0.9457	0.052*	
C5B	0.930 (4)	0.6390 (8)	1.137 (2)	0.048 (5)	
H5BA	1.0715	0.6520	1.1079	0.058*	
H5BB	0.9160	0.6433	1.2471	0.058*	
C6B	0.734 (4)	0.6602 (8)	1.057 (2)	0.050 (5)	
H6B	0.5977	0.6428	1.0517	0.060*	
C7B	0.666 (5)	0.7294 (11)	0.765 (4)	0.098 (12)	
H7BA	0.7893	0.7257	0.6933	0.117*	
H7BB	0.7228	0.7473	0.8460	0.117*	
C8B	0.471 (4)	0.7466 (9)	0.686 (2)	0.064 (6)	
C9B	0.369 (5)	0.7277 (11)	0.557 (3)	0.090 (9)	
H9B	0.4288	0.7044	0.5175	0.109*	
C10B	0.176 (5)	0.7452 (10)	0.492 (3)	0.080 (8)	
H10B	0.1056	0.7328	0.4058	0.097*	
C11B	0.078 (5)	0.7791 (10)	0.541 (3)	0.078 (7)	
H11B	−0.0543	0.7895	0.4908	0.094*	
C12B	0.180 (4)	0.7969 (11)	0.665 (3)	0.082 (8)	
H12B	0.1141	0.8200	0.7020	0.098*	
C13B	0.375 (5)	0.7826 (10)	0.737 (3)	0.076 (7)	
H13B	0.4460	0.7963	0.8204	0.092*	
O1C	0.709 (3)	0.5680 (5)	0.3297 (14)	0.052 (5)	
O2C	0.420 (2)	0.5922 (6)	0.1855 (13)	0.055 (5)	
F1C	0.440 (2)	0.5625 (5)	0.5606 (11)	0.074 (5)	
F2C	0.414 (2)	0.6230 (5)	0.5063 (14)	0.077 (5)	
F3C	0.1583 (19)	0.5832 (5)	0.4231 (9)	0.071 (5)	
C1C	0.382 (3)	0.5872 (8)	0.447 (2)	0.042 (6)	
C2C	0.519 (4)	0.5805 (6)	0.306 (2)	0.033 (5)	
O1D	0.378 (2)	0.4904 (5)	1.2965 (13)	0.047 (4)	
O2D	0.090 (2)	0.5162 (5)	1.1558 (11)	0.045 (4)	
F1D	0.366 (2)	0.5249 (4)	0.9285 (10)	0.054 (3)	
F2D	0.375 (2)	0.4654 (5)	0.9654 (12)	0.071 (4)	
F3D	0.638 (2)	0.5015 (5)	1.0584 (11)	0.066 (4)	
C1D	0.420 (3)	0.4990 (8)	1.0293 (18)	0.037 (4)	
C2D	0.281 (4)	0.5034 (7)	1.1753 (19)	0.036 (6)	

### Atomic displacement parameters (Å^2^)

**Table:**

	*U*^11^	*U*^22^	*U*^33^	*U*^12^	*U*^13^	*U*^23^
O1A	0.045 (10)	0.100 (17)	0.058 (9)	0.007 (10)	0.001 (7)	0.006 (10)
F1A	0.035 (8)	0.079 (12)	0.070 (7)	0.005 (7)	−0.004 (5)	0.024 (8)
F2A	0.097 (10)	0.120 (15)	0.022 (6)	−0.010 (10)	−0.007 (5)	−0.020 (7)
F3A	0.060 (8)	0.051 (10)	0.042 (6)	−0.004 (7)	0.013 (5)	−0.006 (6)
N1A	0.036 (9)	0.043 (10)	0.026 (6)	−0.004 (7)	0.002 (5)	−0.004 (6)
N2A	0.077 (14)	0.070 (17)	0.050 (10)	−0.010 (12)	−0.007 (9)	−0.024 (11)
N3A	0.078 (15)	0.075 (18)	0.053 (11)	−0.007 (12)	−0.012 (10)	−0.010 (11)
N4A	0.090 (16)	0.11 (2)	0.068 (12)	−0.036 (14)	0.005 (11)	−0.046 (13)
C1A	0.033 (12)	0.050 (11)	0.044 (8)	0.005 (8)	−0.002 (7)	0.004 (7)
C2A	0.058 (13)	0.046 (10)	0.028 (8)	−0.006 (8)	−0.001 (8)	0.007 (7)
C3A	0.035 (12)	0.047 (10)	0.020 (8)	−0.009 (8)	−0.003 (7)	−0.005 (7)
C4A	0.055 (12)	0.036 (11)	0.024 (6)	−0.008 (9)	0.000 (6)	−0.006 (7)
C5A	0.046 (11)	0.043 (10)	0.037 (9)	−0.002 (8)	−0.002 (7)	−0.006 (7)
C6A	0.042 (11)	0.043 (12)	0.043 (8)	0.006 (8)	−0.004 (7)	0.003 (8)
C7A	0.067 (17)	0.07 (2)	0.077 (15)	0.031 (16)	0.012 (12)	0.042 (16)
C8A	0.075 (14)	0.075 (16)	0.034 (10)	0.012 (11)	−0.018 (9)	0.019 (9)
C9A	0.091 (16)	0.091 (17)	0.060 (13)	0.011 (12)	−0.005 (10)	0.002 (11)
C10A	0.094 (16)	0.098 (17)	0.053 (12)	0.011 (12)	0.000 (10)	0.013 (12)
C11A	0.093 (15)	0.088 (17)	0.056 (12)	0.016 (12)	−0.014 (10)	0.032 (11)
C12A	0.086 (16)	0.097 (17)	0.070 (14)	0.002 (12)	−0.012 (10)	0.013 (12)
C13A	0.090 (16)	0.084 (17)	0.066 (13)	0.002 (11)	−0.008 (10)	0.004 (11)
O1B	0.068 (10)	0.048 (13)	0.062 (8)	0.003 (9)	−0.020 (7)	0.015 (9)
F1B	0.128 (12)	0.080 (13)	0.030 (6)	−0.003 (10)	0.007 (7)	0.019 (7)
F2B	0.045 (8)	0.072 (11)	0.049 (6)	−0.009 (7)	0.012 (5)	0.017 (7)
F3B	0.100 (11)	0.077 (12)	0.027 (6)	−0.002 (9)	0.018 (6)	−0.001 (7)
N1B	0.030 (9)	0.043 (10)	0.036 (7)	−0.004 (7)	−0.003 (6)	0.004 (6)
N2B	0.067 (11)	0.072 (17)	0.040 (9)	0.004 (11)	−0.004 (9)	−0.023 (10)
N3B	0.062 (12)	0.085 (19)	0.053 (10)	0.007 (11)	0.008 (10)	−0.006 (11)
N4B	0.067 (12)	0.10 (2)	0.078 (13)	0.017 (12)	0.000 (10)	−0.019 (14)
C1B	0.044 (12)	0.052 (12)	0.038 (8)	−0.014 (9)	−0.013 (7)	0.008 (7)
C2B	0.058 (12)	0.050 (11)	0.028 (8)	−0.022 (8)	−0.005 (7)	0.014 (7)
C3B	0.060 (12)	0.051 (12)	0.024 (8)	−0.017 (9)	−0.012 (7)	0.007 (8)
C4B	0.052 (12)	0.047 (11)	0.031 (7)	−0.005 (9)	−0.003 (7)	0.010 (7)
C5B	0.056 (11)	0.050 (10)	0.037 (9)	−0.004 (9)	−0.008 (8)	−0.001 (8)
C6B	0.051 (11)	0.057 (14)	0.042 (8)	−0.008 (9)	−0.006 (7)	0.008 (8)
C7B	0.09 (2)	0.09 (3)	0.11 (2)	−0.034 (19)	−0.037 (16)	0.08 (2)
C8B	0.065 (13)	0.081 (17)	0.046 (10)	0.005 (11)	0.004 (9)	0.020 (10)
C9B	0.098 (15)	0.098 (18)	0.073 (13)	0.031 (13)	−0.027 (11)	−0.004 (12)
C10B	0.091 (15)	0.092 (18)	0.057 (12)	0.018 (12)	−0.010 (10)	0.019 (11)
C11B	0.072 (15)	0.087 (18)	0.075 (13)	0.008 (12)	−0.007 (10)	0.018 (11)
C12B	0.069 (14)	0.100 (18)	0.076 (13)	0.013 (12)	−0.003 (10)	0.004 (12)
C13B	0.071 (14)	0.093 (18)	0.064 (12)	0.008 (11)	−0.003 (10)	0.008 (11)
O1C	0.036 (9)	0.077 (14)	0.043 (7)	0.014 (9)	−0.007 (6)	−0.002 (8)
O2C	0.035 (8)	0.108 (16)	0.022 (7)	−0.011 (9)	−0.005 (6)	0.003 (8)
F1C	0.063 (8)	0.130 (16)	0.028 (6)	0.010 (9)	0.010 (5)	0.020 (8)
F2C	0.085 (10)	0.091 (15)	0.055 (7)	0.010 (10)	0.010 (6)	−0.027 (9)
F3C	0.044 (9)	0.151 (17)	0.019 (5)	0.010 (8)	0.003 (5)	−0.004 (7)
C1C	0.028 (14)	0.08 (2)	0.020 (10)	−0.002 (12)	−0.002 (9)	−0.012 (13)
C2C	0.047 (15)	0.016 (14)	0.038 (12)	−0.010 (11)	0.004 (11)	−0.002 (10)
O1D	0.035 (8)	0.070 (13)	0.037 (7)	0.006 (8)	0.013 (6)	0.012 (8)
O2D	0.028 (9)	0.087 (14)	0.020 (6)	0.020 (8)	0.009 (5)	0.006 (7)
F1D	0.060 (7)	0.089 (9)	0.013 (5)	0.004 (6)	0.003 (4)	0.016 (5)
F2D	0.099 (10)	0.078 (10)	0.038 (6)	0.001 (7)	0.015 (6)	−0.016 (6)
F3D	0.040 (6)	0.116 (13)	0.043 (6)	0.008 (6)	0.003 (5)	0.019 (7)
C1D	0.039 (9)	0.059 (10)	0.012 (8)	0.006 (7)	0.000 (7)	−0.001 (7)
C2D	0.030 (13)	0.050 (17)	0.027 (11)	0.012 (12)	0.000 (9)	−0.001 (10)

### Geometric parameters (Å, º)

**Table:**

O1A—C1A	1.43 (3)	N1B—H1BB	0.9100
O1A—C7A	1.48 (3)	N1B—C4B	1.51 (2)
F1A—C2A	1.37 (2)	N1B—C5B	1.44 (3)
F2A—C3A	1.31 (2)	N2B—N3B	1.24 (3)
F3A—C3A	1.34 (2)	N2B—C6B	1.39 (3)
N1A—H1AA	0.9100	N3B—N4B	1.16 (3)
N1A—H1AB	0.9100	C1B—H1B	1.0000
N1A—C4A	1.48 (2)	C1B—C2B	1.52 (3)
N1A—C5A	1.46 (3)	C1B—C6B	1.57 (3)
N2A—N3A	1.25 (3)	C2B—H2B	1.0000
N2A—C6A	1.50 (3)	C2B—C3B	1.55 (3)
N3A—N4A	1.14 (3)	C3B—C4B	1.43 (3)
C1A—H1A	1.0000	C4B—H4BA	0.9900
C1A—C2A	1.46 (3)	C4B—H4BB	0.9900
C1A—C6A	1.53 (3)	C5B—H5BA	0.9900
C2A—H2A	1.0000	C5B—H5BB	0.9900
C2A—C3A	1.55 (3)	C5B—C6B	1.51 (3)
C3A—C4A	1.55 (3)	C6B—H6B	1.0000
C4A—H4AA	0.9900	C7B—H7BA	0.9900
C4A—H4AB	0.9900	C7B—H7BB	0.9900
C5A—H5AA	0.9900	C7B—C8B	1.45 (4)
C5A—H5AB	0.9900	C8B—C9B	1.42 (4)
C5A—C6A	1.50 (3)	C8B—C13B	1.45 (4)
C6A—H6A	1.0000	C9B—H9B	0.9500
C7A—H7AA	0.9900	C9B—C10B	1.38 (4)
C7A—H7AB	0.9900	C10B—H10B	0.9500
C7A—C8A	1.50 (4)	C10B—C11B	1.38 (4)
C8A—C9A	1.37 (4)	C11B—H11B	0.9500
C8A—C13A	1.40 (4)	C11B—C12B	1.37 (4)
C9A—H9A	0.9500	C12B—H12B	0.9500
C9A—C10A	1.44 (4)	C12B—C13B	1.38 (4)
C10A—H10A	0.9500	C13B—H13B	0.9500
C10A—C11A	1.37 (4)	O1C—C2C	1.21 (2)
C11A—H11A	0.9500	O2C—C2C	1.26 (2)
C11A—C12A	1.26 (4)	F1C—C1C	1.35 (3)
C12A—H12A	0.9500	F2C—C1C	1.35 (3)
C12A—C13A	1.38 (4)	F3C—C1C	1.33 (2)
C13A—H13A	0.9500	C1C—C2C	1.53 (3)
O1B—C1B	1.36 (3)	O1D—C2D	1.27 (2)
O1B—C7B	1.42 (3)	O2D—C2D	1.21 (2)
F1B—C2B	1.35 (2)	F1D—C1D	1.29 (3)
F2B—C3B	1.37 (2)	F2D—C1D	1.31 (3)
F3B—C3B	1.34 (2)	F3D—C1D	1.30 (2)
N1B—H1BA	0.9100	C1D—C2D	1.56 (3)
			
C1A—O1A—C7A	111.5 (17)	O1B—C1B—H1B	108.8
H1AA—N1A—H1AB	107.1	O1B—C1B—C2B	105.9 (15)
C4A—N1A—H1AA	107.8	O1B—C1B—C6B	107.8 (18)
C4A—N1A—H1AB	107.8	C2B—C1B—H1B	108.8
C5A—N1A—H1AA	107.8	C2B—C1B—C6B	116.6 (19)
C5A—N1A—H1AB	107.8	C6B—C1B—H1B	108.8
C5A—N1A—C4A	118.2 (17)	F1B—C2B—C1B	108.4 (18)
N3A—N2A—C6A	113.7 (18)	F1B—C2B—H2B	107.3
N4A—N3A—N2A	177 (3)	F1B—C2B—C3B	108.9 (18)
O1A—C1A—H1A	108.8	C1B—C2B—H2B	107.3
O1A—C1A—C2A	111.7 (16)	C1B—C2B—C3B	117.1 (15)
O1A—C1A—C6A	104.8 (15)	C3B—C2B—H2B	107.3
C2A—C1A—H1A	108.8	F2B—C3B—C2B	107.8 (18)
C2A—C1A—C6A	113.8 (19)	F2B—C3B—C4B	112.1 (15)
C6A—C1A—H1A	108.8	F3B—C3B—F2B	105.8 (18)
F1A—C2A—C1A	111.1 (18)	F3B—C3B—C2B	105.2 (14)
F1A—C2A—H2A	106.5	F3B—C3B—C4B	110 (2)
F1A—C2A—C3A	105.3 (17)	C4B—C3B—C2B	116 (2)
C1A—C2A—H2A	106.5	N1B—C4B—H4BA	108.5
C1A—C2A—C3A	120.3 (17)	N1B—C4B—H4BB	108.5
C3A—C2A—H2A	106.5	C3B—C4B—N1B	115 (2)
F2A—C3A—F3A	108.7 (15)	C3B—C4B—H4BA	108.5
F2A—C3A—C2A	109.5 (16)	C3B—C4B—H4BB	108.5
F2A—C3A—C4A	106.2 (18)	H4BA—C4B—H4BB	107.5
F3A—C3A—C2A	106.6 (17)	N1B—C5B—H5BA	107.1
F3A—C3A—C4A	109.1 (15)	N1B—C5B—H5BB	107.1
C4A—C3A—C2A	116.6 (17)	N1B—C5B—C6B	120.7 (19)
N1A—C4A—C3A	114.0 (17)	H5BA—C5B—H5BB	106.8
N1A—C4A—H4AA	108.8	C6B—C5B—H5BA	107.1
N1A—C4A—H4AB	108.8	C6B—C5B—H5BB	107.1
C3A—C4A—H4AA	108.8	N2B—C6B—C1B	109 (2)
C3A—C4A—H4AB	108.8	N2B—C6B—C5B	109.3 (18)
H4AA—C4A—H4AB	107.7	N2B—C6B—H6B	108.8
N1A—C5A—H5AA	109.4	C1B—C6B—H6B	108.8
N1A—C5A—H5AB	109.4	C5B—C6B—C1B	111.8 (19)
N1A—C5A—C6A	111.3 (16)	C5B—C6B—H6B	108.8
H5AA—C5A—H5AB	108.0	O1B—C7B—H7BA	109.2
C6A—C5A—H5AA	109.4	O1B—C7B—H7BB	109.2
C6A—C5A—H5AB	109.4	O1B—C7B—C8B	112 (2)
N2A—C6A—C1A	107.4 (19)	H7BA—C7B—H7BB	107.9
N2A—C6A—H6A	110.0	C8B—C7B—H7BA	109.2
C1A—C6A—H6A	110.0	C8B—C7B—H7BB	109.2
C5A—C6A—N2A	105.6 (16)	C9B—C8B—C7B	120 (3)
C5A—C6A—C1A	113.6 (16)	C9B—C8B—C13B	119 (2)
C5A—C6A—H6A	110.0	C13B—C8B—C7B	121 (3)
O1A—C7A—H7AA	111.0	C8B—C9B—H9B	121.9
O1A—C7A—H7AB	111.0	C10B—C9B—C8B	116 (3)
O1A—C7A—C8A	103.9 (18)	C10B—C9B—H9B	121.9
H7AA—C7A—H7AB	109.0	C9B—C10B—H10B	117.2
C8A—C7A—H7AA	111.0	C11B—C10B—C9B	126 (3)
C8A—C7A—H7AB	111.0	C11B—C10B—H10B	117.2
C9A—C8A—C7A	121 (3)	C10B—C11B—H11B	121.4
C9A—C8A—C13A	119 (3)	C12B—C11B—C10B	117 (3)
C13A—C8A—C7A	119 (3)	C12B—C11B—H11B	121.4
C8A—C9A—H9A	121.5	C11B—C12B—H12B	118.8
C8A—C9A—C10A	117 (3)	C11B—C12B—C13B	122 (3)
C10A—C9A—H9A	121.5	C13B—C12B—H12B	118.8
C9A—C10A—H10A	120.0	C8B—C13B—H13B	120.5
C11A—C10A—C9A	120 (3)	C12B—C13B—C8B	119 (3)
C11A—C10A—H10A	120.0	C12B—C13B—H13B	120.5
C10A—C11A—H11A	119.4	F1C—C1C—F2C	105.5 (15)
C12A—C11A—C10A	121 (3)	F1C—C1C—C2C	112.4 (19)
C12A—C11A—H11A	119.4	F2C—C1C—C2C	112.3 (19)
C11A—C12A—H12A	119.0	F3C—C1C—F1C	105.9 (17)
C11A—C12A—C13A	122 (4)	F3C—C1C—F2C	106.2 (19)
C13A—C12A—H12A	119.0	F3C—C1C—C2C	114.0 (15)
C8A—C13A—H13A	120.2	O1C—C2C—O2C	130.8 (17)
C12A—C13A—C8A	120 (3)	O1C—C2C—C1C	115.5 (17)
C12A—C13A—H13A	120.2	O2C—C2C—C1C	113.4 (19)
C1B—O1B—C7B	116.5 (19)	F1D—C1D—F2D	106.0 (14)
H1BA—N1B—H1BB	107.4	F1D—C1D—F3D	107.8 (18)
C4B—N1B—H1BA	108.4	F1D—C1D—C2D	112.2 (18)
C4B—N1B—H1BB	108.4	F2D—C1D—C2D	109.7 (19)
C5B—N1B—H1BA	108.4	F3D—C1D—F2D	108.8 (19)
C5B—N1B—H1BB	108.4	F3D—C1D—C2D	112.2 (15)
C5B—N1B—C4B	115.7 (16)	O1D—C2D—C1D	115.4 (17)
N3B—N2B—C6B	113.9 (19)	O2D—C2D—O1D	128.7 (16)
N4B—N3B—N2B	170 (3)	O2D—C2D—C1D	115.6 (15)
			
O1A—C1A—C2A—F1A	145.1 (17)	F1B—C2B—C3B—F3B	−51 (2)
O1A—C1A—C2A—C3A	−91 (2)	F1B—C2B—C3B—C4B	−172.2 (18)
O1A—C1A—C6A—N2A	−71 (2)	F2B—C3B—C4B—N1B	37 (3)
O1A—C1A—C6A—C5A	172.1 (19)	F3B—C3B—C4B—N1B	153.7 (17)
O1A—C7A—C8A—C9A	−70 (3)	N1B—C5B—C6B—N2B	155.3 (19)
O1A—C7A—C8A—C13A	118 (2)	N1B—C5B—C6B—C1B	−84 (3)
F1A—C2A—C3A—F2A	−72.0 (17)	N3B—N2B—C6B—C1B	81 (2)
F1A—C2A—C3A—F3A	170.6 (12)	N3B—N2B—C6B—C5B	−157 (2)
F1A—C2A—C3A—C4A	48.5 (19)	C1B—O1B—C7B—C8B	171 (2)
F2A—C3A—C4A—N1A	−173.5 (17)	C1B—C2B—C3B—F2B	−62 (2)
F3A—C3A—C4A—N1A	−57 (2)	C1B—C2B—C3B—F3B	−174.5 (17)
N1A—C5A—C6A—N2A	148.0 (17)	C1B—C2B—C3B—C4B	64 (3)
N1A—C5A—C6A—C1A	−94 (2)	C2B—C1B—C6B—N2B	−174.0 (19)
N3A—N2A—C6A—C1A	80 (2)	C2B—C1B—C6B—C5B	65 (3)
N3A—N2A—C6A—C5A	−159 (2)	C2B—C3B—C4B—N1B	−88 (2)
C1A—O1A—C7A—C8A	177.4 (19)	C4B—N1B—C5B—C6B	35 (3)
C1A—C2A—C3A—F2A	161.7 (17)	C5B—N1B—C4B—C3B	47 (3)
C1A—C2A—C3A—F3A	44 (2)	C6B—N2B—N3B—N4B	−158 (12)
C1A—C2A—C3A—C4A	−78 (2)	C6B—C1B—C2B—F1B	−169.4 (16)
C2A—C1A—C6A—N2A	166.2 (18)	C6B—C1B—C2B—C3B	−46 (3)
C2A—C1A—C6A—C5A	50 (2)	C7B—O1B—C1B—C2B	−105 (2)
C2A—C3A—C4A—N1A	64 (2)	C7B—O1B—C1B—C6B	129 (2)
C4A—N1A—C5A—C6A	74 (2)	C7B—C8B—C9B—C10B	177 (3)
C5A—N1A—C4A—C3A	−52 (2)	C7B—C8B—C13B—C12B	−176 (3)
C6A—C1A—C2A—F1A	−97 (2)	C8B—C9B—C10B—C11B	0 (5)
C6A—C1A—C2A—C3A	27 (3)	C9B—C8B—C13B—C12B	3 (4)
C7A—O1A—C1A—C2A	−87 (2)	C9B—C10B—C11B—C12B	0 (5)
C7A—O1A—C1A—C6A	148.9 (19)	C10B—C11B—C12B—C13B	1 (4)
C7A—C8A—C9A—C10A	−175 (2)	C11B—C12B—C13B—C8B	−3 (4)
C7A—C8A—C13A—C12A	178 (2)	C13B—C8B—C9B—C10B	−2 (4)
C8A—C9A—C10A—C11A	2 (4)	F1C—C1C—C2C—O1C	−30 (3)
C9A—C8A—C13A—C12A	7 (4)	F1C—C1C—C2C—O2C	155.7 (18)
C9A—C10A—C11A—C12A	−5 (4)	F2C—C1C—C2C—O1C	89 (2)
C10A—C11A—C12A—C13A	9 (5)	F2C—C1C—C2C—O2C	−86 (2)
C11A—C12A—C13A—C8A	−9 (4)	F3C—C1C—C2C—O1C	−150 (2)
C13A—C8A—C9A—C10A	−3 (3)	F3C—C1C—C2C—O2C	35 (3)
O1B—C1B—C2B—F1B	71 (2)	F1D—C1D—C2D—O1D	−154.0 (19)
O1B—C1B—C2B—C3B	−165.6 (17)	F1D—C1D—C2D—O2D	31 (3)
O1B—C1B—C6B—N2B	−55 (2)	F2D—C1D—C2D—O1D	89 (2)
O1B—C1B—C6B—C5B	−176 (2)	F2D—C1D—C2D—O2D	−86 (3)
O1B—C7B—C8B—C9B	−64 (4)	F3D—C1D—C2D—O1D	−32 (3)
O1B—C7B—C8B—C13B	115 (3)	F3D—C1D—C2D—O2D	153 (2)
F1B—C2B—C3B—F2B	61 (2)		

### Hydrogen-bond geometry (Å, º)

Cg1 and Cg2 are the mid-points of the C10*A*—C11*A* and C10*B*—C11*B* bonds, respectively.

**Table:**

*D*—H···*A*	*D*—H	H···*A*	*D*···*A*	*D*—H···*A*
N1*A*—H1*AA*···O1*D*^i^	0.91	1.89	2.75 (2)	156
N1*A*—H1*AB*···O2*D*^ii^	0.91	1.79	2.677 (19)	163
N1*B*—H1*BA*···O1*C*^iii^	0.91	1.76	2.66 (2)	170
N1*B*—H1*BB*···O2*C*^iv^	0.91	1.85	2.75 (2)	167
N1*A*—H1*AA*···O1*C*	0.91	2.57	2.88 (3)	101
N1*A*—H1*AB*···O1*C*	0.91	2.57	2.88 (3)	101
N1*B*—H1*BB*···O2*D*^v^	0.91	2.63	2.94 (3)	101
N1*B*—H1*BA*···O2*D*^v^	0.91	2.70	2.94 (3)	96
N1*A*—H1*AB*···F3*D*^i^	0.91	2.60	3.010 (16)	108
N1*B*—H1*BB*···F3*C*^iv^	0.91	2.55	2.954 (17)	108
C4*A*—H4*AA*···O1*C*	0.99	2.47	3.12 (3)	122
C6*A*—H6*A*···O1*D*^i^	1.00	2.42	3.14 (3)	129
C4*B*—H4*BA*···O2*D*^v^	0.99	2.39	3.08 (3)	126
C6*B*—H6*B*···O2*C*^iii^	1.00	2.37	3.22 (3)	142
C12*B*—H12*B*···N4*A*^vi^	0.95	2.71	3.42 (4)	133
C4*A*—H4*AA*···F3*B*	0.99	2.50	3.29 (3)	137
C4*A*—H4*AA*···F3*C*^v^	0.99	2.69	3.27 (3)	118
C5*A*—H5*AA*···F1*A*	0.99	2.59	3.18 (2)	118
C5*A*—H5*AB*···F3*D*^i^	0.99	2.70	3.31 (2)	120
C6*A*—H6*A*···F3*A*	1.00	2.35	2.88 (2)	112
C4*B*—H4*BA*···F2*A*	0.99	2.55	3.39 (3)	142
C4*B*—H4*BA*···F3*D*	0.99	2.85	3.34 (3)	111
C5*B*—H5*BA*···F2*B*	0.99	2.51	2.95 (2)	107
C7*B*—H7*BA*···F1*B*	0.99	2.59	3.15 (5)	116
C7*B*—H7*BA*···*Cg*1^v^	0.99	2.87	3.73 (4)	146
C7*A*—H7*AA*···*Cg*2^v^	0.99	2.64	3.46 (4)	140

Symmetry codes: (i) *x*, *y*, *z*−1; (ii) *x*+1, *y*, *z*−1; (iii) *x*, *y*, *z*+1; (iv) *x*+1, *y*, *z*+1; (v) *x*+1, *y*, *z*; (vi) −*x*, *y*+1/2, −*z*+1.
